# The Elevator—Its Principle and Utility in Extraction

**Published:** 1893-08

**Authors:** Edward Cox

**Affiliations:** England; Auckland, New Zealand


### The Elevator—Its Principle and Utility in Extraction.

BY EDWARD COX, L.D.S., OF ENGLAND; AUCKLAND, NEW ZEALAND.

In some form or other the elevator is almost as old as the "key" but unlike the "key" never reigned the sole and supreme weapon with the rank and file of dental operators; was, in fact, never popular or in general use. In most surgeries it lay rusting in the pouch, a mere addendum or curiosity, few understanding the principle of its application, and fewer still acquiring confidence and dexterity in its use. It was, and still is, but in diminishing degree, a much abused instrument. This abuse was not deserved. It has, however, survived objections and dislike; it is winning even its opponents, and to the initiated is simply indispensable. Veteran operators who, years ago had become wonderful experts with the key, expressed the same repugnance to the forceps as that evinced by many practitioners, medical and dental, to the elevator. This repugnance, I am convinced, arose and still comes from want of knowledge and experience, from not knowing the varied and best forms to use and how to use them. A very skillful dentist, and a well-known inventor of dental appliances told me he had never used the elevator in his life. I inquired why ? and he replied "Because I am afraid." The same feeling would possess an old operator with the key who refused the forceps for the removal of a lower molar, and putting the fulcrum upon the buccal portion of the alveolus adroitly turns over the tormentor but with an awful crunch.

These venerable and authoritative keepers of the key objected to the forceps (as some now object to the elevator) as a "dangerous instrument," that it gave more pain and required more strength and skill than the established method. This is not surprising. Nothing succeeds like success, and an operator is generally more successful with an instrument he is accustomed to than with one much better which he has never proved. They who had become experts with the key could hardly be persuaded that it was in reality a barbarous instrument compared with its younger and more scientific rival. In one respect only could it claim a general advantage; it required less physical force than the forceps. With the lever power of the claw comparatively slight lateral force was sufficient to remove—if not mountains—molars firm as a rock. Something must give way, something must come, a double portion it might be, leaving a heavy footprint upon the yielding process and mucous membrane. It was a great advance upon this when the attack involved the tooth only. The danger of fracture was greatly reduced and the bruising and cutting of the mucous membrane was avoided when seized and embraced by well-adapted blades pressed steadily downwards, an offending molar was loosened and removed almost independently of the alveolar process. The application of force too could be guided by the apparent or probable inclination of the tooth. Force with the key could only be applied laterally and slightly upward. With the forceps it could be applied in any and every direction, and now the multiplication of patterns adapted to nearly every form of peculiarity and difficulty, places it *par excellence* the instrument for regular daily use. That it requires greater physical force, that in some instances extraction with the forceps tests and exhausts the strength of the operator, must be admitted, (I have only heard this denied by inflated self-conceit) but not infrequently the demand is lessened by the slight leverage gained by the cleavage of the blades between the fangs and process, especially if the fangs prove to be conjoined or conical. Every operator is occasionally agreeably surprised to feel in these conical cases a tooth spring up locked and loose between the blades. It is also notably reduced in those cases where what is known as the "cow-horn" pattern is applicable, in which the leverage principle is utilized by inserting and partially closing the "horns" between the fangs, and so finding a fulcrum in the sides of the crown and the surrounding process.

Here then it may reasonably be asked, With this whole array of modern forceps at hand what is the necessity, what is the principle and value of the Elevator?

Its principle may be said to combine that of the key and forceps, that is, it combines the leverage principle with the application of force directly upon the tooth or fang itself.

The blade of the elevator being carefully placed, force is applied at an obtuse angle, almost in a vertical line upon and against the tooth or fang making the inner edge of the socket a fulcrum, or an adjoining tooth, or sometimes the finger or thumb protected by lint or napkin. This will more clearly appear as we look at the instrument itself, at its most varied and useful patterns and consider their *modus operandi.*

There are operators who confine themselves to some one form which they have proved and value above rubies, for by persistent practice they have become so expert as rarely to fail in most difficult and forlorn cases. A confrere, in the old country, carries about with him a small elevator and assures you: "I can take out anything with this." He is often met with a smile of incredulity, yet those who have seen him operate with it know that this assertion is almost literally true. He uses a short, stumpy, home-made elevator, with ebony or vulcanite handle, rounded and flattened somewhat at the sides, with a straight, half-round, tapering blade, serrated and rounded at its edge. The fact is, practice gives efficiency with almost any kind of instrument and success gives confidence and exclusive preference for the form so approved. The operator who exploits with his one elevator-elect regards any other form superfluous and probably discounts the ability of an operator who requires and prefers a variety. This discount I, for one, am willing to bear, for I have found it advisable in my own practice to have at least five different patterns. These patterns and their applications I will introduce by briefly narrating how I first came to use and appreciate the elevator.

Not long after I commenced practice I was requested to visit a neighboring town and operate for a patient under chloroform. The case was peculiar and difficult. I think I have only known one like it. The patient, a lady, was determined to have all her teeth removed, although most of them were in good condition and capable of long service; they were also very firmly fixed, for she was strong, broadly built with remarkably developed maxillæ. In vain I entreated her to leave most of them in; she had extraction on the brain, and if I would not remove them some one else should. Experience has led me to believe that it is better to forego and lose a patient than operate against personal judgment and conviction. The operation proved more difficult than I could foresee. This arose less from the fact that the teeth were unusually firm and the patient strong and large-boned than from the refusal of the chloroformist to carry anesthesia beyond the excitement stage; in fact, from timidity or unwillingness to produce anesthesia at all. However, despite railing and bellicose resistance I succeeded in extracting all save the fangs of the two upper canines and of two upper bicuspids. These I could not dislodge; so retreated, resolved to renew the assault under different conditions.

Relating the case to my friend, the late Wm. H. Rowe, of Preston, Lancashire. He said, "I should have used the elevator for those remaining fangs." Passing into his operating room he showed me the form he would have used and how to use it, at the same time asking me to take it and try it upon the case in hand. Chloroform was given again, but this time thoroughly, and (as arranged) by an experienced administrator who had been a student under Sir James Simpson. To my delight I removed each of the fangs with the elevator, so kindly lent, and without a second attempt. The patient was quite unconscious and the whole case did not occupy more than twenty minutes. Henceforth the elevator became to me indispensable and of all instruments has been the most useful and unfailing.

The elevator here referred to has a short, tapering shaft fitted into a pear-shaped handle, slightly flattened at the sides and bent about three-quarters of an inch from the end almost at right angles. It is adapted to nearly every case where an elevator is indicated; to broken down upper bicuspids and canines, to the buccal fangs of upper molars (as a rule no elevator is applicable to upper wisdom teeth, I have used but very rarely No. 2 elevator) and to the fangs of lower molars, bicuspids and canines.

Take e. g. a badly decayed right upper bicuspid fang. My usual procedure is as follows:

(*a*) Carefully examine the character and conditions of the case noting any coign of vantage.

(*b*) Apply some form of local anesthetic.

(*c*) Wrap the end of the first finger of the left hand in strong lint or a small, narrow napkin made for the purpose and place it on the palatal surface to support the process as a fulcrum and to receive the blade of the elevator and so protect the tongue, etc.

(*d*) Hold the elevator firmly in the palm of the right hand, extend the index finger along the upper surface to guide and position the blade and aid and direct the application of the force. Gradually slide, press and wriggle the blade down the buccal side of the fang until you judge the firm portion has been reached, then raise the handle towards the cheek until almost in a line with the fang and apply force steadily downwards and inwards increasing as may be necessary, now and again with a lifting tilting motion, at the same time keeping the elevator firmly pressed against the side to direct, support and act as a break to the application. As a rule the offender will be simply levered out of its stronghold with the least possible pain and disturbance, sometimes sent flying instanter, again reluctantly sliding down and lying prostrate against the napkin-covered finger, yet more frequently dismissed presto, the edge of the elevator occasionally digging through or grazing the napkin and if not well protected finding its bed in the ball of the finger where its mark may be seen after many days. This, however, rarely occurs. When it does it is usually in early practice from not knitting and keeping the elbow steadily to the side and moving the body a little in support of the attacking force. The same *modus operandi* applies to the upper canine fangs and to the buccal fangs of upper molars; also when used for the lower jaw save, of course, that the handle is depressed and force directed in a line with the direction of the fangs.

Returning to the upper jaw. Not unfrequently we have the fangs of an upper molar just held together by a remnant of decaying tooth substance with the buccal fangs deeply buried. It is usual and no doubt the best practice to attack this remnant with the fang forceps beginning at the palatal. But the forceps does not always succeed, and when it does, not without great difficulty. I have repeatedly in such cases, and also in cases of fracture with the forceps where the palatal fang is readily perceptible, been rewarded by the use of an elevator similar in principle to the one first described, but longer in the shaft, with round ball handle and the blade end bent at a more obtuse angle. Placing the left index finger with napkin on the buccal side to protect the cheek, and leaning the patient's head somewhat in the direction of the palatal fang, the elevator (No. 2) is passed across the mouth, its blade is pressed down on the palatal fang with wheedling urgency and force applied steadily and tiltingly downwards and outwards. The tooth or root, as a rule, will not be sent flying from its bed but will be turned over as a stubborn, uprooted pine-tree stump and can then be lifted away *ad libitum*.

For an upper wisdom tooth or remnant the elevator is contraindicated, although in a few instances it has proved in my experience admirably useful.

For lower wisdom teeth I formerly used a pair of elevators with lateral blades right and left. I now prefer for most cases the straight spear-pointed elevator. This is an old form but has been modified and improved by Mr. Coleman, of London. This inserted between the wisdom tooth and second molar and sent as a wedge between the wisdom tooth and its socket, making this and the molar a fulcrum, is then used with a twisting, tilting motion in line with the posterior curve of the wisdom fangs until it lifts and turns the offender backwards and over. Often when it is not thrown right back and clear away it is effectually lifted and may be picked away with the forceps and very rarely clinging to the loose posterior mucous membrane.

There is one class of wisdom teeth that cannot be thus treated; cases where from the premature loss of the second molar, or from lack of space owing to the too vertical rise of the maxillary ramus the tooth as it erupts is pressed and tilted forward until it falls upon its face, the masticating surface looking directly at the posterior surface of the second molar, or bending still more right into the interlying mucous membrane. In such cases—confessedly difficult—I have resorted to my No. 4 elevator with half-round finely-tapering blade, using the posterior buccal edge of the second molar as a fulcrum, or if that tooth be absent, placing the thumb or the left index finger (covered with a napkin) in position for that purpose, then lifting the ill-placed wisdom upwards and backwards until curled right over, or so nearly so, that the forceps has only to lift it away.

There yet remains a few cases where the outer surface is hopelessly gone, but a remnant of the inner remains. For these, if upper bicuspids, I have occasionally used the long elevator (No. 2) across the mouth as for upper molar remnants aforesaid. For canine and central incisor fangs similarly decayed I have found the lateral bladed elevator (No. 6) very useful, always guarding the lip by the napkin-covered index finger.

In this imperfect outline of the *principle and application* of the elevator I imply what in fact I have proved by experience, that the elevator has a unique utility and advantage. In remnants of upper molars where the palatal fang is barely or not at all visible, of bicuspids where the inner portion has gone or is decayed to, or below the gum, or where upper canines are badly broken down by decay, by fracture or by attempted extraction and often with leaning lower wisdom teeth. In these, and other cases, it is simply invaluable.

It is withal less liable to accident, less liable to fracture fangs, the fine apex, for instance of a two-fanged upper bicuspid, than the forceps, and as regards pain it is not more, but less painful than the forceps. That accident and injury are possible and have occurred from its use may be safely admitted, but they are less severe and less frequent than have occurred and do occur with the forceps. The most severe results I have known from the use of the elevator were mild in comparison to those that have been, or might be, recorded from the use of the forceps. I conclude, therefore, that to secure and exalt its position in modern dentistry one condition only is required—enlarged knowledge and experience of its value in actual practice.

