# Correction

**Published:** 1893-11

**Authors:** 


### Correction.

In the August number of the DENTAL REGISTER, page 377, is an article on "The Elevator—Its Principle and Utility in Extraction," by Edward Cox, L.D.S., the author's name is *Edwin* Cox instead of *Edward*. And in the same article, on page 382, first line, in the following sentence: "at the same time keeping the elevator firmly pressed against the side to direct, support and act as a break to the application;" it should have been: at the same time keeping the *elbow* (instead of elevator) firmly pressed against the side to direct, support and act as a break to the application.

